# Women’s Attitudes Toward Self-Monitoring of Their Pregnancy Using Noninvasive Electronic Devices: Cross-Sectional Multicenter Study

**DOI:** 10.2196/11458

**Published:** 2019-01-07

**Authors:** Katharina Schramm, Niklas Grassl, Juliane Nees, Janine Hoffmann, Holger Stepan, Thomas Bruckner, Markus W Haun, Imad Maatouk, Markus Haist, Timm C Schott, Christof Sohn, Sarah Schott

**Affiliations:** 1 Department of Gynecology and Obstetrics University Women's Clinic Heidelberg Heidelberg Germany; 2 Department of Gynecology and Obstetrics University Women's Clinic Leipzig Leipzig Germany; 3 Institute of Medical Biometry and Informatics University of Heidelberg Heidelberg Germany; 4 Department of General Internal Medicine and Psychosomatics University Hospital Heidelberg Heidelberg Germany; 5 Frauenarztpraxis Markus Haist & Anja Ritthaler Pforzheim Germany; 6 Centre of Dentistry, Department of Orthodontics and Orofacial Orthopedics University of Tuebingen Tuebingen Germany

**Keywords:** eHealth, fetal monitoring, pregnancy, telemedicine

## Abstract

**Background:**

Pregnancy can be distressing, particularly if expectant mothers are worried about the well-being of their fetus. Consequently, the desire for reassurance and frequent fetal monitoring is often pronounced. Smart wearable devices and telemedicine are promising tools that could assist women in self-monitoring their pregnancy at home, hence disburdening emergency departments (EDs). They present the possibility to clarify the need for urgent care remotely and offer tighter pregnancy monitoring. However, patients’ acceptance of such new technologies for fetal monitoring has not yet been explored extensively.

**Objective:**

This survey aimed to elucidate the attitudes of women toward self-monitoring of their pregnancy using noninvasive electronic devices. The technical details of the proposed devices were not specified.

**Methods:**

A cross-sectional multicenter study was conducted at the departments of obstetrics of the University Hospitals of Heidelberg and Leipzig, Germany. All patients seen in the obstetrics clinic who were above 18 years were offered participation. We designed a survey questionnaire including validated instruments covering population characteristics, issues in current and past pregnancies, as well as attitudes toward self-monitoring of pregnancy with smart devices.

**Results:**

A total of 509 pregnant women with no previous experience in telemedicine participated. Only a small minority of 5.9% (29/493) regarded self-monitoring with wearable devices as an alternative to consulting their physicians. Along these lines, only 7.7% (38/496) strongly believed they would visit the ED less often if such devices were readily available. However, if the procedure were combined with a Web-based telemetric physician consult, 13.5% (66/487) would be highly motivated to use the devices. Furthermore, significantly more women regarded it as an alternative prior to seeing a doctor when they perceived a decline in fetal movements (*P*<.001). Interestingly, women with university degrees had a higher propensity to engage in pregnancy self-monitoring compared with women without one (37% vs 23%; *P*=.001). Of the participants, 77.9% (381/489) would like smart wearable devices to measure fetal heart sounds, and 62.6% (306/489) wished to use the devices on their own. Feedback from a doctor or midwife was also very important in their choice of such devices (61.8%, 301/487 wished feedback). The intended frequency of use differed vastly among women, ranging from 13.8% (65/471) who would like to use such a device several times per day to 31.6% (149/471) who favored once per week at most.

**Conclusions:**

Our results point to a skeptical attitude toward pregnancy self-monitoring among pregnant women. Nevertheless, many women are open to using devices for pregnancy monitoring in parallel to consulting their physician. The intention to use such devices several times daily or weekly, expressed by more than half of the participants, highlights the potential of such technologies.

## Introduction

Health surveillance apps and devices are becoming more and more popular [1-5]. Many companies offer personalized health trackers and market them as lifestyle products suitable for everyday application. Approximately 80% of women in their fertile period in the United States own a smartphone [6], and around 23% of female smartphone owners use mobile health apps [7]. Consequently, nutrition and menstrual cycle monitoring, as well as pregnancy diaries, have become quite common [8]. However, no medically reliable, cost-effective wearable device is on the market for pregnancy self-monitoring. The use of such devices by pregnant women at home could enable closer observation, provide reassurance to concerned expectant mothers, and better identify high-risk patients. Such devices would constitute a milestone for obstetric care [9]. Since electronic health (eHealth) devices bring new challenges in terms of reliability and medicolegal responsibility, the attitude and comfort of both patients and medical staff are highly relevant [10]. More generally, there is still a surprising lack of data surrounding eHealth and mobile health devices despite its widely recognized potential [2,11,12].

Giving pregnant women the possibility to monitor their fetus at home does not only offer advantages for themselves but also for their physicians and midwives. In obstetrics as well as other medical disciplines, urgent and nonurgent emergency visits are increasing [13]. Concerns and worries about the unborn child are common reasons for emergency consultations in obstetrics. In these cases, it is often challenging for patients to judge whether a given condition is pathological or physiological, resulting in avoidable consultations. Several studies indicate that pregnant women are frequently dismissed upon entering the emergency room since no pathological finding was detected despite a high sense of urgency felt by the patient [14,15]. Additional data from prolonged home monitoring could potentially add diagnostic value in such situations. It is also conceivable that in the future, the additional data might enable medical staff to reassure patients and their partners remotely. For this scenario to become a reality, evidence of the advantages of home monitoring would be required, and legal responsibilities would need to be clarified first.

Prolonged or even continuous monitoring of pregnancy with smart wearable devices that assess several fetal parameters could provide a very comprehensive picture of fetal well-being based on an extensive collection of data. Such recordings might, therefore, be of great value for observation and diagnostics. However, women’s willingness to use remote devices at home to monitor pregnancy has hardly been explored. We therefore designed a survey among expectant mothers to elucidate women’s attitudes toward pregnancy monitoring with smart devices.

## Methods

### Survey Design and Questionnaire

Patients from 4 doctors were included in this prospective cross-sectional multicenter survey at the obstetric emergency departments (EDs) of the University Hospital Women’s Clinics in Heidelberg and Leipzig, Germany or cooperating obstetric outpatient clinics. German-speaking women, aged between 18 and 55 years, were eligible to contribute if they showed the capacity for consent and provided informed consent. Approximately 800 eligible patients were offered participation. This accounts for approximately 10% of the obstetrics patients in the participating centers in the inclusion period, and this subset can be considered a random sample. The questionnaire was completed by 509 women, resulting in a participation rate of 63.9% (509/796).

Patients completed the questionnaires pseudonymously on paper while in the clinic. Participants needed approximately 15 minutes for completing the survey and did not receive any compensation. Exclusion criteria consisted of an inadequate understanding of the German language or refusing to participate. The survey was approved by the ethics committees of both medical faculties (Heidelberg S-525/2016, Leipzig 092/17-lk).

The questionnaire consisted of 21 closed-ended questions to allow for quantitative statistical analysis. In addition, we also asked patients to provide their year of birth and their estimated due date. The questions were conceived by 2 experienced obstetricians and a clinical psychologist following a literature review and an interdisciplinary discussion with diverse members of the labor and delivery staff of the University Hospital in Heidelberg. The questionnaire was subsequently tested on 10 volunteers of our target population that were not part of the final study.

The introductory questions captured characteristics of our study population such as their highest education degree, marital status, employment type, and health care plan. These questions were followed by an assessment of their current and previous pregnancies including the number of previous pregnancies, deliveries, previous modes of delivery, planned mode of delivery in this pregnancy, complications in this pregnancy, whether conception was natural, and whether the current pregnancy was a multiple pregnancy. The remaining questions focused on eHealth devices for pregnancy monitoring. The description of the devices in question did not cover technical details but specified that they were noninvasive and that women would be able to put them on autonomously. The first block of these questions inquired under which circumstances and how often the respondent was willing to engage in pregnancy monitoring at home, with possible responses like “if I felt my baby less...” The second block focused on the expectations toward devices to monitor a pregnancy at home such as display of recordings, design, and functionality. The original questions are provided in Multimedia Appendix 1. Further results regarding emergency visits are published elsewhere (Schramm et al, under review).

### Statistical Analysis

The statistical analyses were performed in Microsoft Excel version 15.31 and SAS 9.1 Documentation. The relative frequencies of the replies of close-ended questions were calculated and stated as the percentage of the total numbers of replies. For questions that asked participants to rank their agreement with certain statements on a Likert-scale ranging from 1 to 5, the percentages of replies and the weighted mean were computed (Tables 1 and 2). Inferential statistics comprised chi-square tests for categorical data. For all analyses, statistical significance was at a type 1 error of 5% (2-tailed).

## Results

### Study Population

Sample characteristics are shown in Table 1 and Multimedia Appendix 2. The questionnaire was completed by 509 women, resulting in a participation rate of 63.9% (509/796) for all women that were offered participation. The completeness rate of these data was 96.3% (490/509). The sample is by and large representative of pregnant women in Germany [16], with an overrepresentation of high-risk pregnancies due to the care structure of the participating centers.

### Perception of Telemedicine as Alternative to Physician Consult

In the first step, the questionnaire explored if devices for self-monitoring could reduce physician consultations. When asked whether they regarded self-monitoring of their pregnancy as an alternative to consulting a physician, only 5.9% (29/493) of our study participants strongly agreed, 36.7% (181/493) of participants strongly disagreed, and the remaining 57.4% (283/493) of women favored an intermediate to skeptical standpoint toward this statement (Table 2). However, significantly more women regarded it as an alternative prior to seeing a doctor when they felt fewer baby movements (*P*<.001). Still, only 7.7% (38/496) strongly believed they would visit the ED less often during pregnancy if such devices were in place.

The picture was slightly different if the assumed use of mobile devices to monitor pregnancy were combined with a telemedical consultation with a physician. In this scenario, 13.5% (66/487) of participants strongly believed they would use such devices, and only 12.5% (61/487) categorically rejected that notion (Multimedia Appendix 3). A small minority of patients felt insecure using such technologies if Web-based contact with a physician was established. However, we registered a strong agreement of 41.8% (192/471) to the statement that cardiotocography (CTG) provides more certainty than self-monitoring.

### Expected Properties of Mobile Devices for Pregnancy Monitoring

Preferences for the readout of mobile devices for pregnancy monitoring varied. While 29.8% (134/450) of the participants preferred a simple binary reading stating either that everything is normal or that consulting a physician is recommended, a majority of 39.6% (178/450) were in favor of more detailed information allowing graduating fetal well-being and providing information on fetal status. The remaining 30.7% (138/450) even wanted such devices to display as much information as possible.

**Figure 1:**
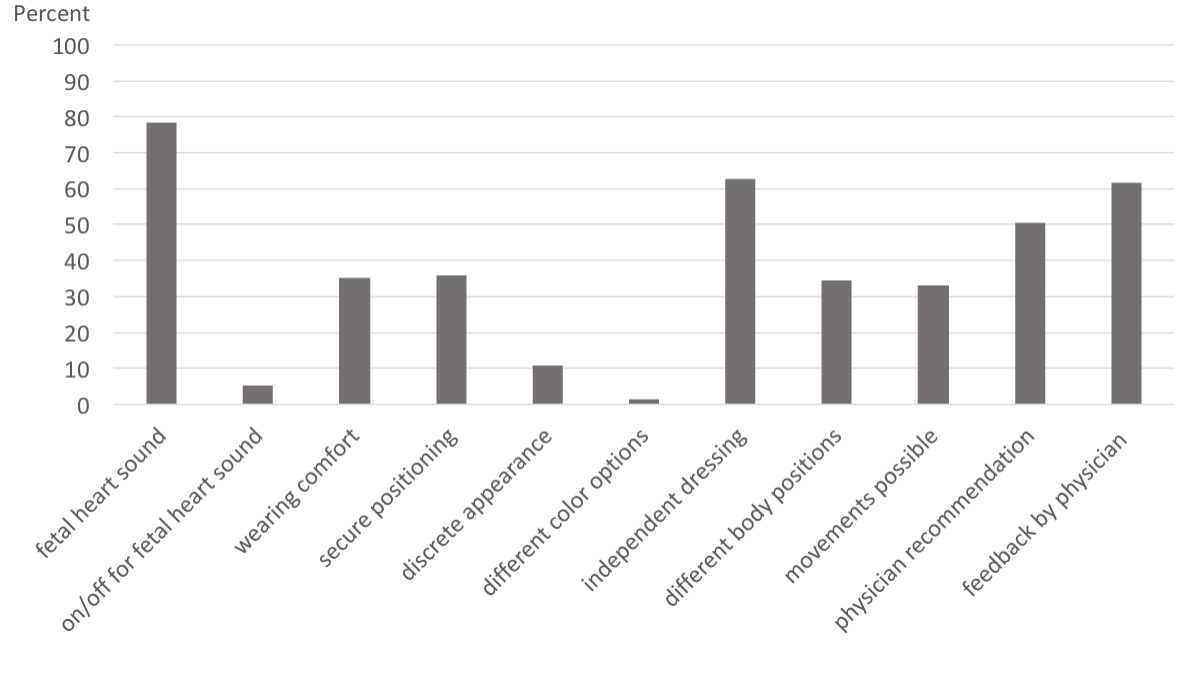
Patient preferences for features of mobile devices for pregnancy monitoring.

The expectations of different features for pregnancy monitoring are summarized in Figure 1. Patients were asked to indicate which features of mobile devices they regarded as particularly important. Results are displayed as the percentage of total replies. Multiple answers were possible.

The most important feature recommended by our study participants was to enable mothers to listen to their baby’s heartbeat (78.2%, 381/487). An independent application, an endorsement by physicians or midwives, and feedback about proper utilization was also important to potential users (62.8%, 306/487; 50.5%, 246/487; and 61.8%, 301/487, respectively). Properties of comparatively less importance to the users included wearing comfort and secure positioning (34.5%, 168/487 and 35.7%, 174/487, respectively). Around 1-third of participants would have liked the devices to allow measurements in different body positions and during movement. The least important features were the possibility to mute the fetal heart sound and the optical design.

Finally, we also addressed how frequently a mobile device for pregnancy self-monitoring would be used. Interestingly, 13.8% (65/471) of the participants indicated that they would perform pregnancy monitoring at home several times per day and 4.8% (23/471) even would use it “all the time if possible.” A frequency of once per day was preferred by 22.1% (104/471). The number of participants who were inclined to use it 1-4 times a week was 27.6% (130/471), and 31.6% (149/471) would use it less than once per week.

The attitudes toward pregnancy monitoring with eHealth devices differed substantially depending on socioeconomic status. Among patients with a university degree, 37.1% (77/207) would have consulted their obstetrician less often if they had the chance to monitor their fetus at home compared with 23.1% (66/286) without a university degree (*P*=.001). At the same time, 75.8% (157/207) of academics preferred a more detailed readout over a binary readout of monitoring devices compared with 66.3% (161/243) of nonacademics (*P*=.03). Both the attitude toward pregnancy monitoring with wearable devices and preferences for features of mobile devices did not depend on age, the number of previous pregnancies, marital status, or health care plan.

**Table 1:** Sample characteristics of the study population.

Characteristic		Responses, n (%)
**Age (years)**		
	18-20	12 (2.4)
	21-25	47 (9.3)
	26-30	168 (33.2)
	31-35	178 (35.2)
	36-40	84 (16.6)
	>41	17 (3.4)
**Highest education degree**		
	Dropped out of school	11 (2.2)
	Secondary education ending with ninth grade	41 (8.1)
	Secondary education ending with tenth grade	147 (29.0)
	University entrance diploma	101 (19.9)
	University degree	207 (40.8)
**Marital status**		
	Married, living with spouse	470 (92.7)
	Married, living separated from spouse	9 (1.8)
	Single, without children	16 (3.2)
	Single, with children	11 (2.2)
	Widowed	1 (0.2)
**Employment**		
	Full-time (>35 hours per week)	143 (28.3)
	Part-time (15-34 hours per week)	68 (13.4)
	By the hour (1-14 hours per week)	1 (0.2)
	Educational training (student)	20 (4.0)
	Housewife	41 (8.1)
	Unemployed	14 (2.8)
	Leave of absence (ie, maternity leave)	219 (43.3)
**Health care plan**		
	Public health care	391 (89.9)
	Private health care	44 (10.1)
	Public family health care	43 (9.9)
	Supplementary insurance	29 (6.7)

**Table 2:** Pregnancy monitoring at home as an alternative to direct physician consultation.

If I had the possibility to monitor your baby at home…	Score=1^a^	Score=2	Score=3	Score=4	Score=5	Weighted mean^b^
...I would regard this as an alternative to consulting a physician.	181 (36.7)	102 (20.7)	118 (23.9)	63 (12.8)	29 (5.9)	2.3
...I would regard this as an alternative prior to visiting a physician if I felt my baby less.	132 (26.8)	82 (16.7)	102 (20.7)	112 (22.8)	64 (13.0)	2.8
...I cannot imagine this and would always prefer a direct consult with a physician or midwife.	48 (9.8)	60 (12.2)	115 (23.4)	126 (25.7)	141 (28.8)	3.5
...I would visit the emergency department less often.	120 (24.1)	110 (22.2)	117 (23.6)	111 (22.4)	38 (7.7)	2.7

^a^Participants were asked to indicate their agreement to the following statements on a scale from 1 to 5 (1 signifies strong disagreement and 5 strong agreement); absolute numbers are shown, and percentages or replies are indicated in brackets.

^b^Weighted means are shown for each statement.

The number of pregnancies did not have any impact on the willingness to use eHealth devices prior to seeing a physician (44% in first pregnancy vs 42% in women with previous pregnancies, *P*=.77). Also, we did not detect any significant differences between the attitude toward the use of eHealth devices between patients that had several emergency visits during their pregnancy compared with patients that had no or just 1 emergency visit (47% vs 52%, *P*=.71).

## Discussion

### Principal Findings

Our comprehensive study provides a detailed account of patients’ attitude toward pregnancy monitoring with eHealth devices when faced with several scenarios. A large proportion of women are open to the idea of monitoring their pregnancy using eHealth devices but prefer to use them in addition to rather than instead of consultations with their physician. The data indicate that patients that have never used eHealth devices can imagine consulting a physician using these new technologies. However, the skepticism toward these eHealth devices is greatest in scenarios where these tools are employed to replace a doctor’s consult. This is in line with results from a study on telemedicine in postoperative care that found patients to be afraid of losing their personal relationship with their doctor when engaging in telemedicine [17]. If a scenario is offered to monitor with a device combined with a Web-based consultation with a physician, significantly more patients would feel comfortable using it. Many patients might also underestimate the extent to which telemedicine has already become part of professional medical practice [18].

Also, 36.7% (181/493) of our cohort could imagine using eHealth devices prior to visiting their doctor if they felt fewer baby movements. It seems that a physician’s or midwife’s judgment is regarded as a lot more trustworthy than the reading of a monitoring device. However, it has been well established that face-to-face interactions are not superior to telemedical interactions on professional practice and health care outcomes in several medical specialties [19-21].

High trust in the opinion of a health care professional is also reflected in the features of pregnancy monitoring devices that are most important to patients. Recommendation by a physician or midwife and their feedback about the proper use of the device are found to be very important. Only the ability to record the fetal heart rate and to apply the device independently was discovered to be more essential to patients. All in all, the functionality of such devices is the key to patients compared with design aspects like different color options or discrete appearance. More women with university degrees prefer a detailed over a binary readout of such devices compared with nonacademics. In general, this seems to be the population that feels most inclined to engage in pregnancy monitoring at home. A possible explanation might be that women with a university degree feel more comfortable about autonomous fetal monitoring or expect greater benefits from the use of new technologies. Of note, the proportion of participants with university degrees in our study was 40.8% compared with 14.8% in the general population [22].

### Limitations

The study population was by and large representative of pregnant women in Germany, with a slight overrepresentation of academics and high-risk pregnancies due to the fact that participants were mainly seen at university hospitals with a maximal level perinatal care. Given that high-risk pregnancies imply more extensive monitoring and more frequent antenatal consultations, this patient population is likely to benefit the most from telemedical pregnancy monitoring [23].

Furthermore, this study was carried out by only a few doctors in service. Therefore, not all possible women meeting the inclusion criteria were reached but rather a random cross-section thereof. Hence, we expect selection bias to be limited, especially as this was a bicentric study.

The study is an account of attitudes toward telemedicine in obstetrics at a time when telemedicine is barely used. In interpreting the findings of this study, one has to bear in mind that all study participants had no prior experience with remote pregnancy monitoring. Thus, the application of eHealth devices and telemedicine was left to women´s imagination. Consequently, our study focuses on intent rather than actual behavior. Previous studies suggest that knowledge of telemedicine in the general population is limited, and people who are not familiar with it tend to reject it [24,25]. We assume that the experience and more widespread use of such devices will have a profound impact on those attitudes.

### Outlook for Pregnancy Self-monitoring

The fact that almost 20% of women would be willing to wear devices for pregnancy monitoring all the time or several times daily highlights the desire of some women to closely monitor their pregnancy. In fact, monitoring frequencies of once per week or more—preferred by 2-thirds of our participants—seem only practical using self-monitoring devices. Hence, these responses highlight pregnant women’s desire to get frequent updates on fetal well-being and their self-evolvement. The desired monitoring frequencies require the new devices to be handy and noninvasive. So, far, the CTG does not open options for that kind of monitoring, as it requires a health care professional to set it up correctly. In addition, it is considered an invasive procedure that exposes the fetus to ultrasound. Whether permanent fetal monitoring via CTG causes fetal harm has not been investigated yet, but experts in ultrasound medicine recommend following the as low as reasonably achievable principle for the use of ultrasound in obstetrics [26,27]. Finally, the reading of CTGs has a high intra- and interrater variability [28]. These results fueled the development of computer-based CTG analysis [29] to increase the reliability of CTG interpretation in the future.

Consequently, pregnancy monitoring devices could gain great popularity, though as a supplement rather than as a replacement for pregnancy monitoring by physicians. Nonetheless, it should also be remembered that many women are opposed to extensive monitoring of their pregnancy and the medicalization of the female body [30]. Other disciplines have proven that the acceptance increases with knowledge and a more widespread use [31-35]. Whether such devices will be able to provide reliable diagnoses in the future that are equally as trustworthy and reassuring as the judgment of a physician remains to be elucidated. Prospective studies are needed to address feasibility, safety issues, and effectiveness. For the time being, our study highlights that self-monitoring devices have the potential to become a valuable supplement in antepartum care. However, from a current standpoint, it seems unlikely that devices for pregnancy self-monitoring will relieve EDs from consultations by pregnant women for nonurgent indications any time soon.

### Conclusions

Our study provides a first comprehensive picture of the attitudes of women toward pregnancy self-monitoring at a time when the use of such technology is not established in Germany. The majority of study participants seem reserved toward any form of pregnancy monitoring that does not involve close interactions with health care professionals. However, at the same time, a vast majority expressed interest in frequent fetal monitoring if reliable and easy-to-use devices were available. This suggests that devices for fetal self-monitoring could become a valuable supplement to physicians’ and midwives’ obstetrics care and ought to be investigated in clinical studies soon.

## Appendix

Multimedia Appendix 1 English translation of survey questions.

Multimedia Appendix 2 Patient characterisitcs with respect to previous pregnancies and deliveries.

Multimedia Appendix 3 Pregnancy monitoring with online consultation of physician.

## Abbreviations

CTG: cardiotocography
ED: emergency department
eHealth: electronic health
