# Supplementary material for: Women’s Attitudes Toward Self-Monitoring of Their Pregnancy Using Noninvasive Electronic Devices: Cross-Sectional Multicenter Study
Source: JMIR Mhealth Uhealth. 2019 Jan 7;7(1):e11458. doi: 10.2196/11458 (PMC6329419; doi:10.2196/11458)
Supplement: Multimedia Appendix 1 [file mhealth_v7i1e11458_app1.pdf]

1: Items of the questionnaire addressing telemedicine

No	item
Q1	Date of birth
Q2	Expected due date of pregnancy
Q3	What is your highest educational degree?<br>Quit school<br>Hauptschulabschluss (Nine years of schooling)<br>Realschulabschluss (Ten years of schooling)<br>Abitur (thirteen years of schooling)<br>University degree
Q4	Marital status<br>married, living with spouse<br>married, separated from spouse<br>single without children<br>single with children<br>widowed
Q5	Employment status<br>Full time (>35 hours/week)<br>Part time (15-34 hours/week)<br>By the hour (1-14 hours/week)<br>Educational training (student)<br>Housewife<br>Unemployed<br>Leave of absence (i.e. maternity leave)
Q6	Healthcare plan<br>Public healthcare<br>Private healthcare

	Public family healthcare<br><br>Supplementary insurance

	If I had the opportunity to monitor my unborn child at home…
Q7	……..I would regard this as an alternative to reduce scheduled obligatory visits to an obstetric care department. (Likert-Scale)
Q8	……..I would regard this as an alternative to urgent consultation, if I sense less fetal movements. (Likert-Scale)
Q9	……..I can't imagine using such devices: I always prefer face-to-face contact with my healthcare provider. (Likert-Scale)
Q10	……..and get feedback from a midwife/nurse/obstetrician, I would regard this as an alternative to reduce scheduled obligatory visits in obstetrics. (dichotomic)
Q11	……..and get feedback from a midwife/nurse/obstetrician, I would regard this as an alternative to urgent consultation if I sense less fetal movements. (dichotomic)
Q12	……..and get feedback from a midwife/nurse/obstetrician, I can't imagine using such devices and would always prefer primary contact to my doctor or midwife. (dichotomic)
Q13	……..my emergency visits would decrease. (Likert-Scale)
Q14	……..using a mobile device (medical device plus application) and consulting doctors online, I think I cannot imagine such developments. (Likert-Scale)
	If there was the opportunity to monitor my pregnancy home, using a mobile device (medical device plus application), and consulting doctors online, I think: …
Q15	……A CTG with a doctor appears safer to me. (Likert-Scale)
Q16	……I feel uncertain with the usage of such technology. (Likert-Scale)
Q17	……I could envision such technology. (Likert-Scale)
Q18	……I would definitely use such technology. (Likert-Scale)
Q19	How frequent would you apply such a device if it was home based?<br>ideally all the time<br>several times a day<br>once a day<br>1-4x per week<br>less often

Q20	How detailed should the provided information be?<br>simple output such as “everything ok” vs “physician consultation recommended”<br>grading with details specifying situation of the baby<br>as much information as possible
Q21	What features of a likewise device would be of special importance to you? (multiple answers allowed)<br>hearing my baby’s heartbeat<br>possibility to switch the audio of my baby’s heartbeat on or off<br>wearing comfort<br>secure positioning<br>discrete appearance of device<br>different color options of device<br>independent dressing possible<br>recording in different body positions possible<br>recommendation by my physician<br>feedback by a physician about proper application
