# Supplementary material for: Women’s Attitudes Toward Self-Monitoring of Their Pregnancy Using Noninvasive Electronic Devices: Cross-Sectional Multicenter Study
Source: JMIR Mhealth Uhealth. 2019 Jan 7;7(1):e11458. doi: 10.2196/11458 (PMC6329419; doi:10.2196/11458)

Supplementary figure 1: Patient characterisitcs with respect to previous pregnancies (left) and deliveries (right).

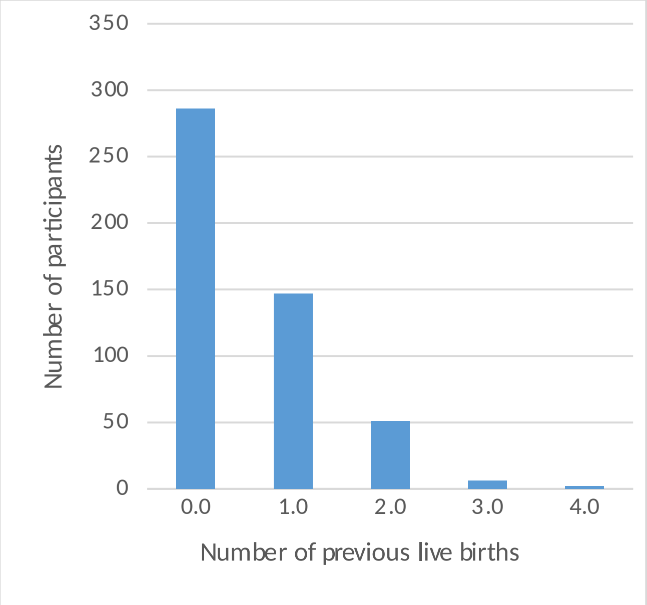

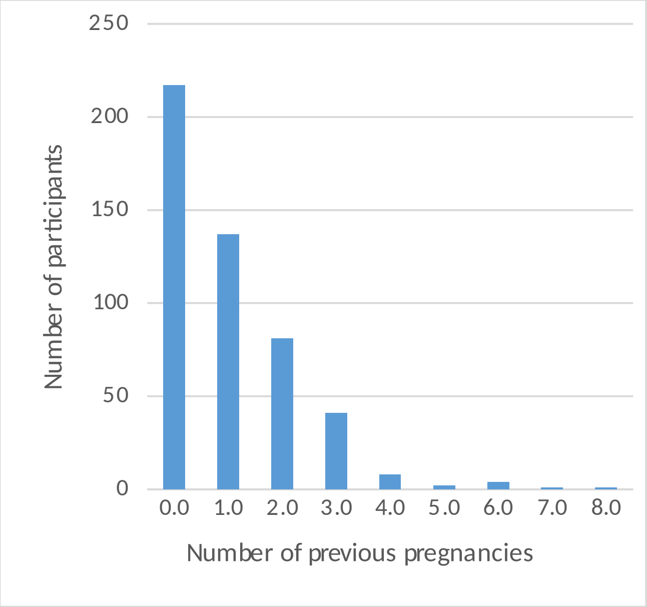

Supplement: Multimedia Appendix 2 [file mhealth_v7i1e11458_app2.pdf]
