# Supplementary material for: Women’s Attitudes Toward Self-Monitoring of Their Pregnancy Using Noninvasive Electronic Devices: Cross-Sectional Multicenter Study
Source: JMIR Mhealth Uhealth. 2019 Jan 7;7(1):e11458. doi: 10.2196/11458 (PMC6329419; doi:10.2196/11458)
Supplement: Multimedia Appendix 3 [file mhealth_v7i1e11458_app3.pdf]

Supplementary table 1: Pregnancy monitoring with online consultation of physician
Participants were asked to indicate their agreement to the following statements on a scale from 1 to 5 where 1 signifies strong disagreement and 5 signifies strong agreement. Absolute numbers are shown and percentages indicated in brackets as well as weighted means.

**The possibility to monitor my pregnancy with a mobile device and consult a physician online provokes the following thoughts.**	**1**	**2**	**3**	**4**	**5**	**Weighted mean**
I could imagine to use such devices	57 (11.7)	87 (17.9)	127 (26.1)	154 (31.7)	61 (12.5)	3.2
I would definitely use such devices	66 (13.6)	105 (21.6)	156 (32.0)	99 (20.3)	61 (12.5)	3.0
I feel insecure using these technologies	77 (16.1)	116 (24.3)	142 (29.8)	94 (19.7)	48 (10.1)	2.8
Cardiotocography at the physician provides more certainty	23 (4.9)	65 (13.8)	104 (22.1)	130 (27.6)	149 (31.6)	3.8
